# Anti-apoptotic effect of claudin-1 in tamoxifen-treated human breast cancer MCF-7 cells

**DOI:** 10.1186/1471-2407-10-548

**Published:** 2010-10-12

**Authors:** Harue Akasaka, Fuyuki Sato, Satoko Morohashi, Yunyan Wu, Yang Liu, Jun Kondo, Hiroki Odagiri, Kenichi Hakamada, Hiroshi Kijima

**Affiliations:** 1 Department of Pathology and Bioscience, Hirosaki University Graduate School of Medicine, Hirosaki 036-8562, Japan; 2 Department of Pathology, College of Basic Medical Sciences, China Medical University, Shenyang, 110001, China; 3 Department of Surgery, Hirosaki University Graduate School of Medicine, Hirosaki 036-8562, Japan

## Abstract

**Background:**

Claudin-1 is a membrane protein of tight junctions, and is associated with the development of various cancers. However, the significance of claudin-1 expression in cancer cells is not well understood. Here, we showed for the first time the anti-apoptotic effect of claudin-1 in human breast cancer MCF-7 cells.

**Methods:**

Human breast cancer MCF-7 and T47 D cells were treated with or without tamoxifen, siRNA against claudin-1, or tamoxifen and claudin-1 siRNA. The samples were analyzed by RT-PCR, Western blotting or immunofluorescent staining.

**Results:**

The expression of claudin-1 was upregulated in tamoxifen-treated MCF-7 cells, whereas the expression of claudin-1 was not altered in tamoxifen-treated T47 D cells. Knockdown of claudin-1 by siRNA increased the amount of poly (ADP-ribose) polymerase (PARP) regardless of tamoxifen treatment in MCF-7 cells, but not T47 D cells. In the cell membranes of the MCF-7 cells, tamoxifen treatment increased the amount of claudin-1, but decreased the amount of β-catenin. Claudin-1 siRNA increased the amount of E-cadherin in the cytoplasm of the MCF-7 cells as well as the amount of β-catenin in their cell membranes.

**Conclusion:**

These results indicate that claudin-1 has anti-apoptotic effects, and is involved in the regulation of the expression and subcellular localization of β-catenin and E-cadherin in MCF-7, but not T47 D cells.

## Background

Breast cancer is the second most common cause of female mortality in United States. The breast cancer incidence and mortality rates were about 190,000 and 40,000, respectively, in 2009 [1]. The majority of breast cancers are sporadic, and most risk factors for the disease are related to estrogen exposure. This suggests that insufficient apoptosis in cancer cells is involved in their survival as insuffcient apoptosis leads to the development of chemotherapy resistance and carcinogenesis [2].

Tamoxifen is one of most widely used anti-estrogen drugs for the treatment of human breast cancer [3]. Tamoxifen treatment leads to a rapid decrease in number of S-phase cells, an accumulation of cells in the G1-fraction [4], and the induction of apoptosis *in vivo *and *vitro *[5-7]. Tamoxifen induces apoptosis through several distinct pathways including a mitochondria-dependent pathway, the induction of c-Myc, the activation of members of the mitogen-activated protein kinases (MAPK) family, and the upregulation of p53 [7-11]. However, the detailed molecular mechanisms by which tamoxifen induces apoptosis are not well understood.

Tight junctions and adherens junctions proteins, including claudins, E-cadherin, β-catenin, and ZOs proteins, are responsible for the maintenance of epithelial cell-cell adhesion and defining cell polarity, and are also involved in cell signaling events [12]. Changes in claudin expression are also involved in invasion, metastasis, and colony formation in various cancer cells [13-15]. In a previous study, the mRNA expression of claudin-1 was decreased in the tumor group compared with the control (normal) group in breast cancer tissues [16]. Decreased expression of claudin-1 was also correlated with breast cancer recurrence [17]. However, the relationship between claudin-1 and chemotherapy is poorly understood.

In the present study, we investigated the relationship between claudin-1 and tamoxifen treatment in human breast cancer MCF-7 and T47 D cells. The expression of claudin-1 was upregulated by tamoxifen treatment in MCF-7 cells. Combination treatment with both claudin-1 siRNA and tamoxifen significantly increased the amount of cleaved PARP. Knockdown of claudin-1 affected the expression and subcellular localization of β-catenin and E-cadherin in MCF-7 cells. Our results suggest that claudin-1 has an anti-apoptotic effect, involving the regulation of β-catenin and E-cadherin, in MCF-7 cells.

## Methods

### Cell culture and treatment

MCF-7 and T47 D cells were obtained from the American Type Culture Collection (ATCC, Manassas, VA, USA). These cells were cultured in Dulbecco's Modified Eagle's Medium-high glucose (Sigma Chemical Co., St. Louis, MO, USA) supplemented with 10% fetal bovine serum at 37°C in a humidified atmosphere of 95% air and 5% CO_2_. When the MCF-7 cells were treated with 40 μM of tamoxifen (Sigma) for 20 h, apoptotic reactions were detected as described below. However, the incubation with 40 μM of tamoxifen for more than 24 h resulted in the severe toxicity to cells, and more than 90% of cells were detached from the plates (data not shown). Therefore, we treated the cells with 40 μM of tamoxifen for 20 h in the follow experiments. In addition, we treated MCF-7 cells with 1, 10 or 20 μM of tamoxifen for 48 h in some experiments to observe the longer effects.

### Reverse transcription-polymerase chain reaction (RT-PCR) and real-time PCR

Total RNA was isolated using an RNeasy RNA isolation kit (QIAGEN, Hilden, Germany). First-strand cDNA was synthesized from 1 μg of total RNA using ReverTra Ace (TOYOBO, Osaka, Japan). RT-PCR was performed using an aliquot of first-strand cDNA as a template under standard conditions with Taq DNA polymerase (QIAGEN). The primers were designed to perform optimal RT-PCR by DNASIS software, and the primers used were as follows: claudin-1-F: 5'-CAGCTGTTGGGCTTCATTCTC-3', claudin-1-R: 5'-ATCACTCCCAGGAGGATGCC-3'; claudin 4-F: 5'-ATGGCCTCCATGGGGCTACA-3', claudin 4-R: 5'-AGCGAGTCGTACACCTTGCA-3'; E-cadherin-F: 5'-ACATTGTCACCTCGCAGAC-3', E-cadherin-R: 5'-GCGGATTGTAGAAGTCTTGG-3'; GAPDH-F: 5'-CCACCCATGGCAAATTCCATGGCA-3', GAPDH-R: 5'-AGACCACCTGGTGCTCAGTGTAGC-3'. The amplified products of claudin-1, claudin-4, E-cadherin, and GAPDH were 277 bp, 208 bp, 336 bp, and 696 bp, in length, respectively. The cDNA for claudin-1, claudin-4, E-cadherin, and GAPDH were amplified for up to 25 cycles. The PCR products were separated on 1.5% (w/v) agarose gels.

The real-time PCR was carried out using SYBER Green Master Mix (Applied Biosystems, Tokyo, Japan). The primers used as follows: claudin-1-F: 5'-AGATGAGGATGGCTGTCATTGG -3', claudin-1-R: 5'-CATGCTGTGGCAGCTAAAATAGC-3'; E-cadherin-F: 5'-ACATTGTCACCTCGCAGAC-3', E-cadherin-R: 5'-GCGGATTGTAGAAGTCTTGG-3'; 18 S rRNA-F: 5'-GTAACCCGTTGAACCCCATT-3', 18 S rRNA-R: 5'-CCATCCAATCGGTAGTAGCG-3'. The amplified products of claudin-1, E-cadherin, and 18 S rRNA were 72 bp, 336 bp, and 150 bp, in length, respectively.

### Short interference RNA (siRNA)

Short interference RNA (siRNA) against claudin-1 were synthesized by QIAGEN. The sequences for the sense and anti-sense claudin-1 siRNA were 5'-r (GCAUGGUAUGGCAAUAGAA) d (TT) -3' and 5'-r (UUCUAUUGCCAUACCAUGC) d (TG) -3', respectively. We also used another siRNA against claudin-1 (claudin-1 siRNA2). The sequences for the sense and anti-sense claudin-1 siRNA2 were 5'-r (CGAAAUUGUUACAAUAGAA) d (TT)-3' and 5'-r (UUCUAUUGUAACAAUUUCG) d (TT)-3'. The negative control (scrambled) siRNA sequences were 5'-r (UUCUCCGAACGUGUCACGU) d (TT)-3' and 5'-r (ACGUGACACGUUCGGAGAA) d (TT)-3'. For the siRNA transfection experiments, MCF-7 and T47 D cells were seeded at 5 × 10^4 ^cells per 35-mm well. Twenty-four h later, the siRNA were transfected into the cells using the Lipofectamine RNA iMAX reagent (Invitrogen, Carlsbad, CA, USA). After transfection, the cells were incubated for 48 h and subjected to various analyses.

### Western Blotting

The cells transfected with siRNA were lysed using M-PER lysis buffer (PIERCE, Rockford, IL, USA). Protein concentrations were determined using the bicinchoninic acid (BCA) assay. The obtained lysates (10 μg protein) were subjected to SDS-PAGE, and the acquired proteins were transferred to PVDF membranes (Immobilion P, Millipore, Tokyo, Japan). The membranes were then incubated with antibodies specific for claudin-1 (1:10,000), claudin-4 (1:20,000), and claudin-7 (1:2,000), which were purchased from Invitrogen; E-cadherin (1:1,000), which was purchased from Takara, Shiga, Japan; β-catenin (1:30,000), Bcl-2 (1:2,000), and p21 (1:1,000), which were purchased from EPITOMICS, CA, USA; cyclin D1 (1:1,000), which was purchased from Merck, Darmstadt, Germany; PARP (1:1,000) and cleaved caspase-8 (1:10,000), which were purchased from Cell Signaling Technology, Inc Danvers, MA, USA; Bax (1:1,000), which was purchased from Santa Cruz, CA, USA; p53 (1:2,000), which was purchased from Abcam, Cambridge, UK; and actin (1:30,000) (Sigma), followed by treatment with horseradish peroxidase-conjugated secondary antibody (IBL, Gunma, Japan). Can Get Signal Immunoreaction Enhancer Solution 1 (TOYOBO) was used to dilute the primary antibody. The ECL, ECL-plus, or ECL-advance Western Blotting Detection System (Amersham, Uppsala, Sweden) was used for detection. The intensity of the bands was quantified by using the National Institute of Health Image computer program. The signal intensities were compensated by actin as internal controls.

### Immunofluorescent staining

MCF-7 and T47 D cells were seeded on a 4-chamber slide glass and incubated overnight. The cells were then washed with phosphate-buffered saline (PBS) and fixed with ice-chilled methanol for 30 min, before being permeabilized with 0.2% Triton-X-100 in PBS for 30 min. The permeabilized cells were then washed in PBS twice and treated with 5% normal horse serum in PBS for 30 min (to minimize the non-specific adsorption of antibodies), before being incubated with anti-claudin-1 (1:200), anti-β-catenin (1:300), or anti-E-cadherin (1:300) antibodies at 4°C overnight. The cells were then incubated for 1 h with goat anti-rabbit IgG antibody conjugated to Alexa 488 dye (Molecular Probes, Inc, Tokyo, Japan), while nuclear staining was performed using 4', 6-diamidino-2-phenylindole (DAPI) or Hoechst 33258. Hoechst 33258 staining was used to examine nuclear condensation. The cells were visualized using confocal laser scanning microscopy (Zeiss, LSM 710, Wetzlar, Germany), and the number of cells that were intensely stained with Hoechst 33258 was counted.

## Results

### Tamoxifen treatment induces apoptosis and upregulates the expression of claudin-1 in MCF-7 cells

**Figure 1:**
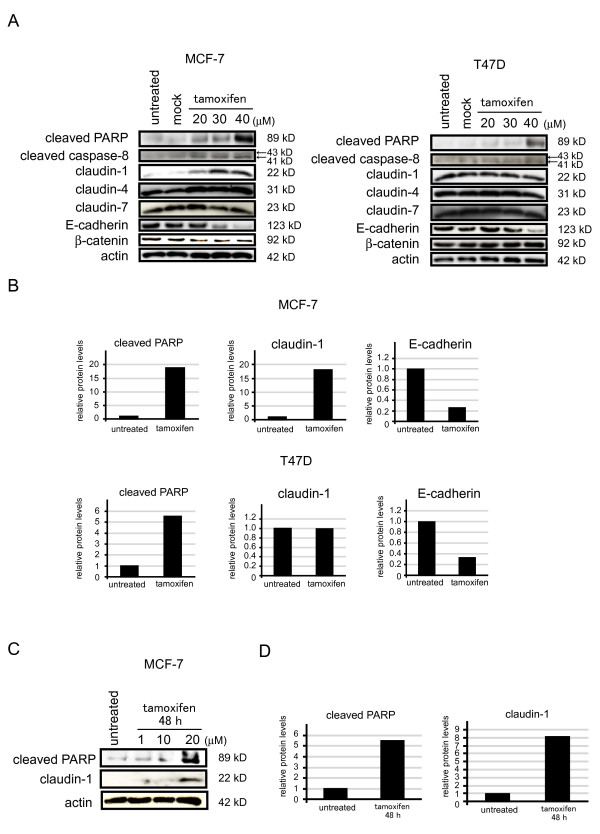
**The protein levels of claudin-1 were increased in MCF-7 cells treated with tamoxifen**. (A) MCF-7 and T47 D cells were treated with the control treatment (buffer alone: Mock) for 20 h or 20, 30, or 40 μM of tamoxifen for 20 h, cell lysates were prepared and subjected to Western blot analyses for cleaved PARP, claudin-1, claudin-4, claudin-7, E-cadherin, cleaved caspase-8, and actin. One representative of at least three independent experiments with similar results is shown. (B) The intensity of the bands for cleaved PARP, claudin-1 and E-cadherin in untreated and tamoxifen (40 μM) - treated cells were quantified. (C) MCF-7 cells were treated with or without 1, 10 or 20 μM of tamoxifen for 48 h, cell lysates were prepared and subjected to Western blot analyses for cleaved PARP, claudin-1 and actin. One representative of at least three independent experiments with similar results is shown. (D) The intensity of the bands for cleaved PARP and claudin-1 in untreated and tamoxifen (40 μM) - treated cells were quantified.

**Figure 2:**
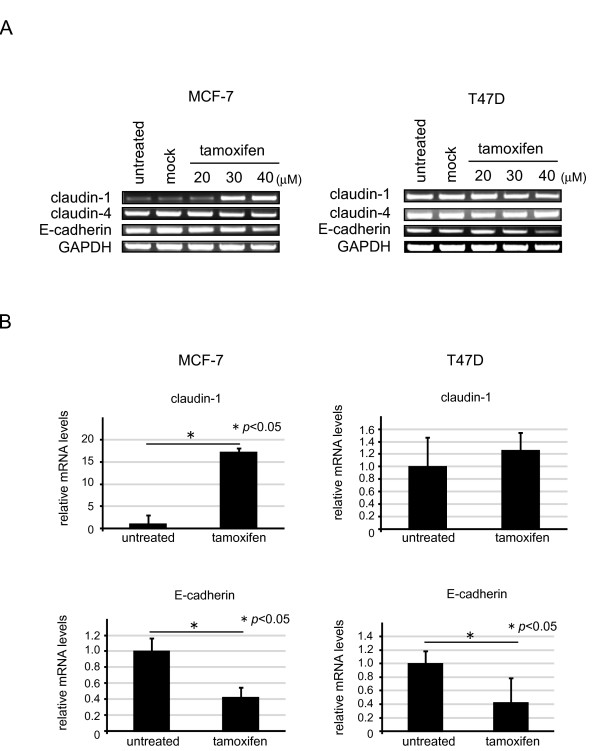
**The mRNA levels of claudin-1 were increased in MCF-7 cells treated with tamoxifen**. (A) After the MCF-7 and T47 D cells had been treated with various concentrations of tamoxifen for 20 h and subjected to RT-PCR analyses. One representative of at least three independent experiments with similar results is shown. (B) MCF-7 and T47 D cells were treated with or without 40 μM of tamoxifen for 20 h, and subjected to real-time PCR analyses for claudin-1 and E-cadherin. Each value represents the mean + SE (*bars*) of three independent experiments *p < 0.05, according to the *t*-test.

We investigated the endogenous expression of claudin-1 in two breast cancer cell lines by Western blotting. The endogenous protein expression of claudin-1 was weak in MCF-7 cells, whereas it was abundantly expressed in T47 D cells (Figure 1A and 1B). In addition, E-cadherin was abundantly expressed in both MCF-7 and T47 D cells. Next, we examined the relationship between tamoxifen and claudin-1. We examined how the protein expression of claudin-1 was affected by tamoxifen treatment. MCF-7 and T47 D cells were treated with various concentrations of tamoxifen for 20 h. Cell lysates were prepared from cells and subjected to Western blot analysis. The protein expression of claudin-1 was slightly increased by treatment with 20 μM tamoxifen in MCF-7 cells, and the cells treated with 30 or 40 μM of tamoxifen treatment showed clear increases in their claudin-1 protein levels. Twenty, 30, or 40 μM of tamoxifen treatment also increased the amounts of cleaved PARP and caspase-8 in the MCF-7 cells, but decreased their expression of β-catenin. The protein expression of E-cadherin was decreased in MCF-7 cells after treatment with 30 or 40 μM of tamoxifen. We also examined whether the longer exposure to tamoxifen affects the expression of claudin-1 in MCF-7 cells. The expression of claudin-1 and the amount of cleaved PARP were significantly increased in the cells treated with 20 μM of tamoxifen for 48 h (Figure 1C and 1D). In T47 D cells, the amounts of cleaved PARP and caspase-8 were increased, whereas the expression of E-cadherin was decreased after 40 μM of tamoxifen treatment. However, the protein expression levels of claudin-1, claudin-4, claudin-7, and β-catenin in T47 D cells were almost unaffected by tamoxifen treatment. We also examined whether the expression of claudin-1 mRNA was affected by tamoxifen treatment. MCF-7 and T47 D cells were treated with various concentrations of tamoxifen for 20 h, and RNA samples were prepared from the cells and subjected to RT-PCR and real-time PCR analyses for claudin-1, claudin-4, E-cadherin, glyceraldehyde-3-phosphate dehydrogenase (GAPDH), and 18 S rRNA (Figure 2A and 2B). The mRNA levels of claudin-1 were significantly increased in MCF-7 cells after treatment with 30 or 40 μM of tamoxifen, whereas tamoxifen treatment did not affect the expression of claudin-1 in T47 D cells. The mRNA expression of E-cadherin was decreased in both MCF-7 and T47 D cells after treatment with 40 μM of tamoxifen. On the other hand, tamoxifen treatment did not affect the expression of claudin-4 in MCF-7 or T47 D cells.

**Figure 3:**
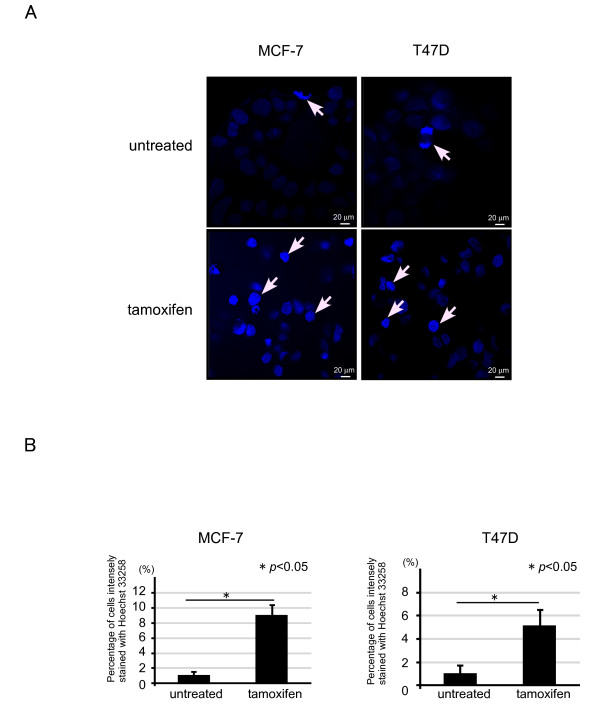
**Nuclear condensation was detected by tamoxifen treatment both in MCF-7 and T47 D cells**. (A) MCF-7 and T47 D cells were seeded in a 4-chamber slide glass and incubated overnight. The cells were treated with or without 40 μM of tamoxifen for 20 h, fixed, and stained with Hoechst 33258. The arrows show nuclear condensation. (B) Percentage of cells intensely fragmented and condensed in nuclei with Hoechst 33258 were counted as positive cells. About 100 total cells were counted in individual ten random microscopic fields at ×40 magnification. Each value represents the mean + SE (*bars*) of two independent experiments *p < 0.05, according to the *t*-test.

Nuclear condensation is one of the features of apoptosis. Using immunofluorescent staining, we examined whether tamoxifen treatment induces nuclear condensation. MCF-7 and T47 D cells were treated with tamoxifen, fixed, and then stained with Hoechst 33258. Nuclear condensation was increased about 9 or 5-fold in MCF-7 or T47 D cells, respectively, treated with 40 μM of tamoxifen compared with that in the untreated-control cells (Figure 3A and 3B).

### Claudin-1 has anti-apoptotic effects under tamoxifen treatment in MCF-7 cells

**Figure 4:**
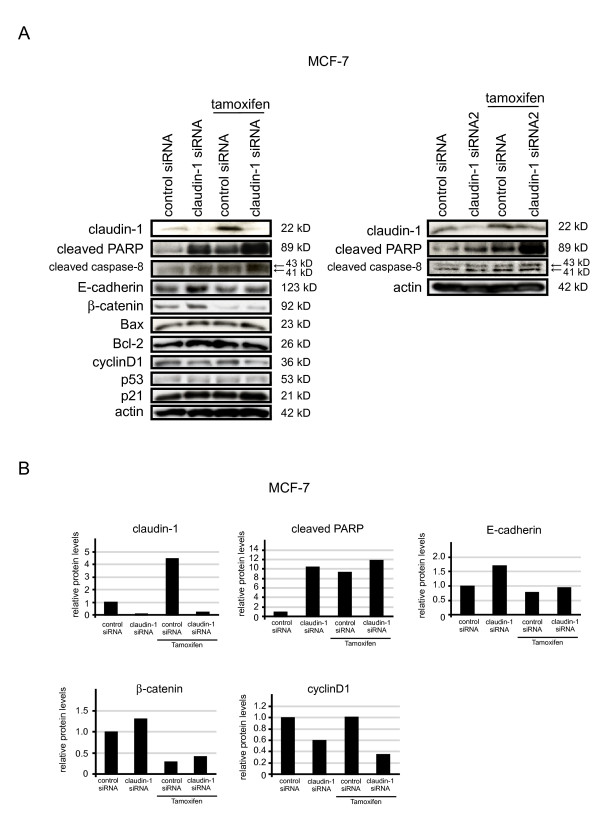
**Anti-apoptotic effect of claudin-1 induced by tamoxifen in MCF-7 cells**. (A) MCF-7 cells were transfected with control siRNA or siRNAs against claudin- 1 (left: claudin-1 siRNA, right: claudin-1 siRNA2) and incubated for 48 h, before being treated with or without 40 μM of tamoxifen and then incubated for a further 20 h. Cell lysates were prepared from the cells and subjected to Western blot analyses for claudin-1, cleaved PARP, cleaved caspase-8, E-cadherin, β-cadherin, Bax, Bcl-2, cyclinD1, p53, p21, and actin. One representative of at least three independent experiments with similar results is shown. (B) The intensity of the bands for claudin-1, cleaved PARP, E-cadherin, β-cadherin and cyclinD1 in control siRNA and claudin-1 siRNA - treated cells with or without tamoxifen (40 μM) for 20 h were quantified.

To understand the mechanism of the upregulation of claudin-1 expression by tamoxifen treatment, we examined whether the transfection of claudin-1 siRNA affected the expression of factors related to apoptosis. MCF-7 cells were transfected with control siRNA or siRNA against claudin-1. After 48 h of transfection, the cells were treated with 40 μM of tamoxifen for 20 h. The cell lysates were then subjected to Western blot analyses for claudin-1, cleaved PARP, cleaved caspase-8, E-cadherin, β-catenin, Bax, Bcl-2, cyclinD1, p53, p21, and actin (Figure 4A and 4B). Claudin-1 knockdown by siRNA significantly reduced the expression of claudin-1 with or without tamoxifen treatment, and the transfection of claudin-1 siRNA increased the amounts of cleaved PARP and caspase-8 with or without tamoxifen treatment. The expression of E-cadherin was upregulated by claudin-1 knockdown without tamoxifen treatment, while the expression of E-cadherin in the presence of claudin-1 siRNA and tamoxifen treatment was slightly increased. On the other hand, the expression of cyclinD1 was downregulated by claudin-1 knockdown with or without tamoxifen treatment, whereas tamoxifen treatment did not affect the expression of cyclinD1. The expression of β-catenin was upregulated by claudin-1 knockdown in the absence of tamoxifen treatment, but combination treatment involving claudin-1 siRNA and tamoxifen had little effect the expression of β-catenin. The expression levels of Bax, Bcl-2, p53, p21, and actin were not affected by claudin-1 knockdown, tamoxifen treatment or combination treatment involving claudin-1 siRNA and tamoxifen.

**Figure 5:**
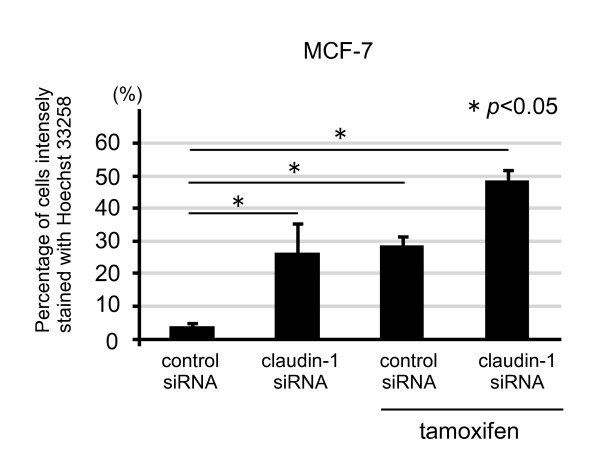
**Nuclear condensation was increased in claudin-1 siRNA treatment of MCF-7**. MCF-7 cells were treated as above, and then stained with Hoechst 33258. Percentage of cells intensely fragmented and condensed in nuclei with Hoechst 33258 were counted as previously described, and each value represents the mean + SE (*bars*) of two independent experiments *p < 0.05, according to the *t*-test.

As nuclear condensation was induced in the cells treated with tamoxifen (Figure 3), we examined whether claudin-1 siRNA treatment had similar effects. Nuclear condensation was increased about 7-fold in the MCF-7 cells treated with claudin-1 siRNA as well as the control siRNA and tamoxifen-treated cells compared with that in the control (only control siRNA treatment) cells (Figure 5). Combination treatment involving claudin-1 siRNA and tamoxifen increased nuclear condensation about 16-fold compared with that in the control cells.

**Figure 6:**
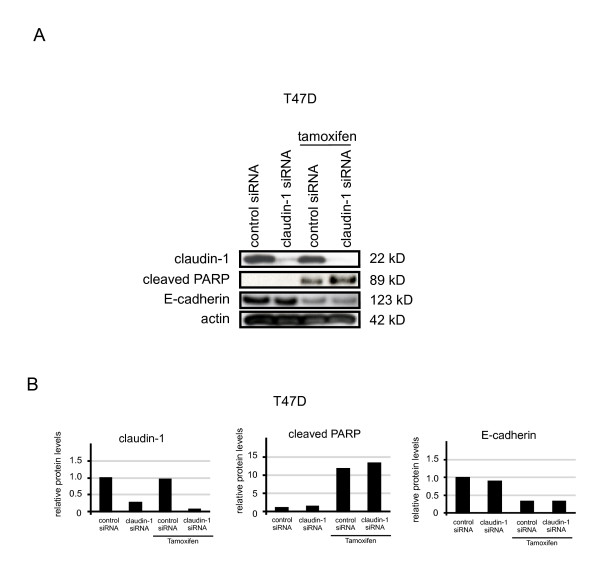
**Claudin-1 knockdown has little effects on apoptosis in T47 D cells**. (A) T47 D cells were treated with control siRNA or siRNA against claudin-1 and treated as above. Cell lysates were subjected to Western blot analyses for claudin-1, cleaved PARP, E-cadherin, and actin. One representative of at least three independent experiments with similar results is shown. (B) The intensity of the bands for claudin-1, cleaved PARP and E-cadherin in control siRNA and claudin-1 siRNA - treated cells with or without tamoxifen (40 μM) for 20 h were quantified.

Next, we examined whether claudin-1 knockdown by siRNA affected the amount of cleaved PARP in T47 D cells. The amount of cleaved PARP was upregulated by tamoxifen treatment, but it was little affected by claudin-1 siRNA treatment or combination treatment involving claudin-1 siRNA and tamoxifen (Figure 6A and 6B).

### Changes in the subcellular localization of E-cadherin and β-catenin in MCF-7 cells treated with claudin-1 siRNA or tamoxifen

**Figure 7:**
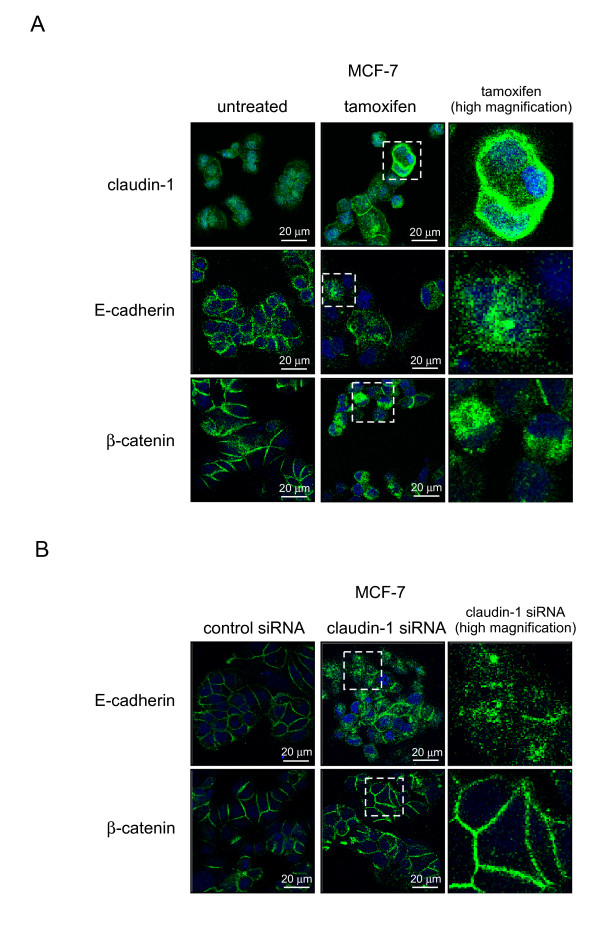
**Subcellular localization of claudin-1, E-cadherin, and β-catenin in MCF-7 cells after tamoxifen or claudin-1 siRNA treatment**. (A) MCF-7 cells were treated with or without 40 μM of tamoxifen for 20 h, fixed, incubated with anti-claudin-1, E-cadherin or β-catenin antibody, and visualized using Alexa488-conjugated secondary antibody (Green). The cells were also counterstained with DAPI (Blue) in order to localize the nucleus. A merged image that is representative of at least two independent experiments with similar results is shown. White dot box for high magnification were also shown. (B) MCF-7 cells were transfected with siRNA against claudin-1. After 48 h incubation, the cells were fixed, incubated with anti-E-cadherin or β-catenin antibody, and visualized using Alexa488. One representative merged image is shown.

**Figure 8:**
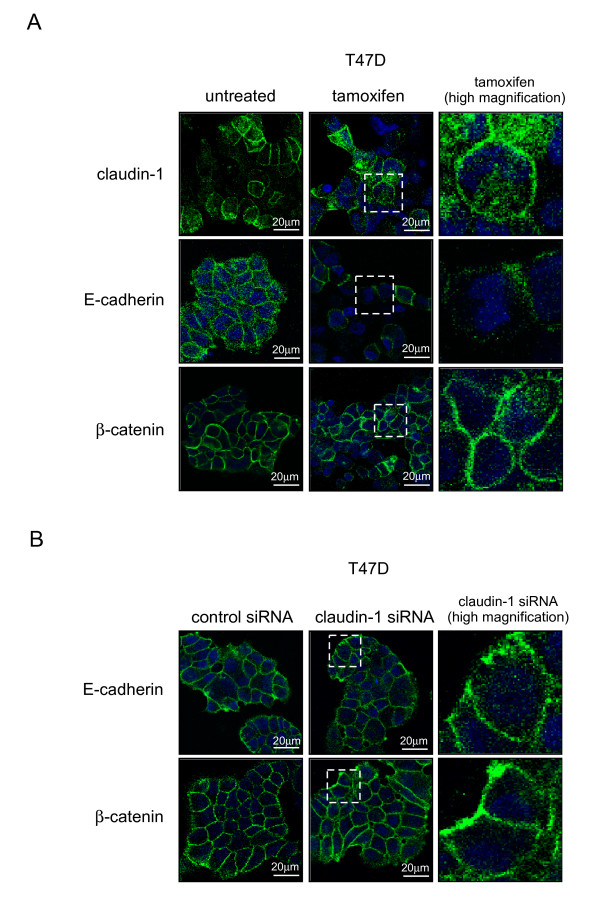
**Subcellular localization of claudin-1, E-cadherin, and β-catenin in T47 D cells after tamoxifen or claudin-1 siRNA treatment**. (A) T47 D cells were treated with or without 40 μM of tamoxifen for 20 h, incubated with anti-claudin-1, E-cadherin, or β-catenin antibody, and visualized using Alexa488. One representative merged image is shown. (B) T47 D cells were transfected with siRNA against claudin-1. After 48 h incubation, the cells were fixed, incubated with anti-E-cadherin or β-catenin antibody, and visualized using Alexa488. One representative merged image is shown.

We investigated whether the subcellular localization of E-cadherin and β-catenin was affected by claudin-1 siRNA or tamoxifen treatment using immunofluorescent staining. As shown in Figure 7A and 8A, tamoxifen treatment increased the amount of claudin-1 in the cell membranes of MCF-7 cells, while tamoxifen treatment did not affect the amount of claudin-1 in T47 D cells. Tamoxifen treatment decreased the amounts of E-cadherin in the cell membranes of MCF-7 and T47 D cells, while it increased the amount of E-cadherin in the cytoplasm of MCF-7, but not T47 D, cells. On the other hand, tamoxifen treatment decreased the amount of β-catenin in the cell membranes of MCF-7 cells, but increased it in the cytoplasm. The amount of E-cadherin in the cell membrane was decreased in MCF-7 cells treated with claudin-1 siRNA, while the amount of E-cadherin in the cytoplasm was increased (Figure 7B). Knockdown of claudin-1 also increased the amount of β-catenin in the cell membranes of MCF-7 cells. In T47 D cells, claudin-1 siRNA treatment did not affect the amount of E-cadherin or β-catenin in the cell membrane or cytoplasm (Figure 8B). These data demonstrated that claudin-1 has anti-apoptotic effects during tamoxifen treatment, which involve changes in the subcellular localization of claudin-1, E-cadherin, and β-catenin in MCF-7 cells.

## Discussion

In this study, we focused on the functions of claudin-1 in human breast cancer cells. Claudins are generally located in the cell membrane and mainly contribute to cell-cell adhesion [18,19]. It was confirmed that claudin-1 is localized to the cell membrane in T47 D cells. However, little claudin-1 was localized to the cell membrane in MCF-7 cells. Tamoxifen treatment increased claudin-1 protein expression as well as its membrane localization in MCF-7 cells, whereas tamoxifen treatment did not affect the expression or subcellular localization of claudin-1 in T47 D cells. Thus, the function of claudin-1 may differ among different cell types. It has been reported that MCF-7 cells have wild type p53 but lack caspase-3. On the other hand, T47 D cells express caspase-3 but p53 is mutated [20,21]. They showed that the sensitivity of these cells against anti-cancer drugs such as staurosporine and Triphala are different. The differential expression of claudin-1 may be also related to differences in phenotype of these two cell lines.

Recent studies have shown the relationship between claudin expression and cellular resistance in tumors [22,23]. The elevated claudin-1 expression induced by 5-fluorouracil (5-FU) or TNF-α treatment is associated with the regulation of apoptosis in nasopharyngeal carcinoma and pancreatic cancer cells, although these cells low levels of protein expression and claudin-1 localization in the membrane were also observed [23,24]. In addition, knockdown of claudin-6 induces cellular resistance to apoptosis in MCF-7 cells [22]. These observations and our findings suggest that the upregulation of claudin-1 by apoptosis-inducers contributes to cellular resistance to apoptosis when claudin-1 protein is expressed at low levels and mislocalized to the cell membrane. However, it is unclear how claudin-1 is regulated by apoptosis inducers. We found that tamoxifen treatment increased the expression of claudin-1 mRNA and proteins related to apoptosis in MCF-7 cells. We speculate that tamoxifen treatment regulates the transcription of claudin-1. Further studies are needed to interpret whether tamoxifen treatment regulates claudin-1 expression.

Next, we investigated the molecular mechanisms of the apoptosis induced by claudin-1 knockdown in MCF-7 cells. Unfortunately, apoptosis-related proteins, such as Bax, Bcl-2, p53, and p21, were not affected by claudin-1 knockdown with or without tamoxifen treatment. However, the expression of cyclin D1 was downregulated by claudin-1 knockdown, regardless of tamoxifen treatment in MCF-7 cells. We speculate that the regulation of apoptosis by claudin-1 knockdown may be related to pathways other than the p21, p53, and mitochondrial-pathways. Lee et al. showed that claudin-1 has anti-apoptotic effects under 5-FU treatment, but they could not demonstrate the molecular mechanisms of claudin-1 induced apoptosis [23].

Interestingly, it has been reported that changes in the subcellular localization of β-catenin or E-cadherin may be related to the regulation of apoptosis [25-28]. 2-methoxyestradiol induces β-catenin expression in prostate cancer cells, but blocks β-catenin degradation, as well as its cytoplasmic or nuclear accumulation, resulting in cell cycle arrest and apoptosis [29]. Therefore, we performed immunofluorescent staining to analyze the changes in the subcellular localization of β-catenin and E-cadherin induced by claudin-1 knockdown or tamoxifen treatment. As expected, claudin-1 knockdown affected the subcellular localization of β-catenin and E-cadherin in MCF-7, but not T47 D cells. Tamoxifen treatment also affected the subcellular localization of β-catenin and E-cadherin. So, we speculate that knockdown of claudin-1 upregulates the protein expression of β-catenin and changes its subcellular localization in MCF-7 cells and then induces cell cycle arrest, resulting in apoptosis. However, tamoxifen treatment downregulates the expression of β-catenin in MCF-7 cells. According to these results, we suggest that tamoxifen treatment upregulates the expression of claudin-1 and that the upregulation of claudin-1 subsequently downregulates the expression of β-catenin. β-catenin may be one of the downstream factors of claudin-1 in MCF-7 cells. However, the detailed mechanism by which claudin-1 regulates the expression of β-catenin needs to be clarified.

We also examined whether other claudins are affected by tamoxifen treatment. The expression of claudin-4 and claudin-7 was not affected by tamoxifen treatment in MCF-7 and T47 D cells as shown in Figure 1A and 2A. Thus, only claudin-1 in claudin's family would be specifically affected by tamoxifen treatment, although we could not elucidate the specific effect of claudin-1 by tamoxifen treatment.

In the present study, we demonstrated the function of claudin-1 in human breast cancer MCF-7 cells. Claudin-1 has anti-apoptotic effects in tamoxifen-treated MCF-7 cells.

## Conclusion

We demonstrated the function of claudin-1 in human breast cancer MCF-7 cells. Our results showed for the first time that claudin-1 has anti-apoptotic effects in tamoxifen-treated MCF-7 cells, involving the regulation of apoptosis-related factors and subcellular localization of adherens junctions.

Our data would be useful for future studies in order to establish the mechanisms of apoptosis regulation in human breast cancer. Further research needs to be clarified the relationship between tight junctions and apoptosis.

## Abbreviations

RT-PCR: Reverse transcription-polymerase chain reaction; PARP: poly (ADP-ribose) polymerase; 5-FU: 5-fluorouracil; TNF-α: tumor necrosis factor-alpha; PBS: phosphate-buffered saline

## Competing interests

The authors declare that they have no competing interests.

## Authors' contributions

HA performed major experimental work. FS designed HA's original work and helped HA's work and completed manuscript. KH and HK corrected HA's manuscript. SM, YW, YL, JK and HO helped HA's work. All authors read and approved the final manuscript.

## Pre-publication history

The pre-publication history for this paper can be accessed here:

http://www.biomedcentral.com/1471-2407/10/548/prepub
